# Medical and Philosophical Intelligence

**Published:** 1818-07

**Authors:** 


### MEDICAL AND PHILOSOPHICAL INTELLIGENCE.

THE *Medical Benevolent Society.*—On Friday, May 18th, the second anniversary of this Institution was held at the Freemason's Tavern; His Royal Highness the DUKE of SUSSEX, having been pleased to become the Patron of the Society, condescended to take the chair. He was supported by the Earls of Rosslyn and Blessington, the President of the Royal College of Physicians (President of the Society), Mr. Cline (Vice President), and by many of the most distinguished members of the profession, as well as by several non-professional gentlemen, who attended as friends to so excellent a charity. The company consisted of about ninety persons. The cloth being drawn, the illustrious Duke made a most eloquent and appropriate address, in which he exhibited a very intimate knowledge of the principles on which the society was founded, and the objects it was intended to embrace. He dwelt, with great feeling, on the necessity which existed for an institution for the relief of the members of the faculty; who were, from a variety of causes, more liable to the vicissitudes of life, and the frowns of adverse fortune, than those of any other profession. And, although there were many societies established to extend pecuniary aid to the widows and orphans of medical men, yet, he believed there was none which, like this, was instituted for the support of the meritorious, but unfortunate and distressed, practitioners themselves. He congratulated the society on the great accession of members, who were doubled in the course of the last year; nor did he doubt, when he considered how numerous a body the medical practitioners throughout the kingdom formed, and that they were men of education and reflection, that, when they became more generally acquainted with this laudable undertaking, they would unanimously give it their support. Most cordially approving, as he did, the views of the worthy President of the College of Physicians, Dr. Latham—the founder, and of those gentlemen who had associated with him in establishing this society, no exertion on his part would be wanting to lend it every possible encouragement; and he trusted that, when he met the members of it at the next anniversary, he should find that its present prosperous aspect would be fully realized, and its benefactors and subscribers be increased in a degree commensurate with the importance of the services of every class of the medical profession.

The Earls of Rosslyn and Blessington severally addressed the meeting; the former, especially, in the warmest terms, passed an elegant and just eulogium on the merits of the medical character. None, (he said,) were more ready, at all times, and to the utmost of their means, to alleviate the afflictions of mankind than the medical practitioner; and the very nature of his services rendered it almost impossible for him to provide adequately against misfortune and the wants of old age. For those, therefore, who were always forward in relieving others, he hoped a call on the affluent would not be made in vain, but that he should see the present institution, receiving the support of all ranks, one of the most flourishing this kingdom had to boast of.

The treasurer announced the receipt of liberal donations from His Royal Highness and the two Noble Lords; 50l. from Richard Thomson, Esq. M.P.; 50l. from Astley Cooper, Esq.; ten guineas from Edward Foster, Esq.; besides many other smaller benefactions. A great number of new members were likewise put in nomination.

---

*Associated Apothecaries and Surgeon-Apothecaries of England and Wales.*—The annual meeting, agreeably to the resolution passed in August 1817, of this association, is to be held at the Crown and Anchor Tavern in the Strand, on Wednesday, July 15th, 1818, at two o'clock, to receive the report of the committee, and make such further arrangements as may tend to promote the public welfare, and, at the same time, support the interest and respectability of the profession. JAMES PARKINSON, Chairman.

Chair to be taken at three o'clock precisely.

The anniversary dinner will be on the same day, at the above tavern, at five o'clock. Such gentlemen as may be disposed to favour the stewards with their company, are requested to signify their intentions on or before Saturday, the 11th inst. by letter, directed to the secretary, Mr. Powell, No. 62, Newman-street, Oxford-street.

*Stewards*—James Upton, Esq.; James Parkinson, Esq.; John Hunter, Esq.; Charles Hayden, Esq.; James Seaten, Esq; Joseph Hayes, Esq.; Walter Drew, Esq.; Edward Leese, Esq.

Tickets 16s. each. *June* 25, 1818.

---

*Case of Poison.*—Mr. Shipman was sent for to a female who, an hour before, had taken arsenic: she was in great pain, and almost senseless: the quantity taken was supposed to be a tea-spoonful. Half a drachm of sulphate of zinc in solution was administered; after the operation of which, he gave the solution of Castile soap, in the proportion of ℥ viij. to a gallon: half of this she took in the course of half an hour, during which time it was occasionally administered in the form of an enema. The pain abated for a short time, but again returned: inflammation appearing to increase, she was bled to the quantity of ℥ xxiv: the symptoms then gradually subsided, and she slept nearly half an hour. At five o'clock, she began to take five grains of sulphuret of potash, with a scruple of magnesia, every four hours. This she continued two days, when symptoms of enteritis again returning; she was bled to ℥ xviij. enemas were administered, and the saline medicine in a state of effervescence: the symptoms subsided; since which, she has been occasionally attacked with similar disturbances in the alimentary canal, but in a more slight degree. She is now perfectly well. On the third day several substances were thrown up from the stomach, which seem to have been coagulated lymph, given out by this organ during its inflammatory state.

---

*Russian Remedy for Hydrophobia.*—Take a good-sized root of the alisma plantago, and two or three small ones; pound or reduce them into a very fine powder, which spread on a piece of buttered bread, and give it to the patient. Two doses, or three at most, are sufficient to eradicate the virulence of the poison, be it ever so violent, or the patient in the worst state, so as to be afraid of water. The efficacy of this root cures also animals bitten by mad dogs, and mad dogs themselves. During the last 25 years, this remedy has not once failed, but has uniformly been a sure means of restoring every person to health, without any bad consequences afterwards, —even those who, from the violence of the poison, rushed on the people and bit them: which facts are particularly ascertained by the government of Tsola.

The plant may be gathered during the whole of the summer; but it operates more efficaciously, if gathered at the latter end of August. The roots of it, being washed clean from mud or earthy matter, must be dried.—*Journal of Science and the Arts*, No. 9.

---

As some distant towns are engaged in illuminating with gas, we copy the following from the *Philosophical Magazine.*

It is sufficiently known, that the production of carburetted hydrogen obtained from coal, and its fitness for the purpose of illumination, varies much according to the circumstances under which the gas is obtained, and the means employed for purifying it. To deprive coal-gas of that portion of sulphuretted hydrogen, with which it is always more or less contaminated, it has hitherto been made to act on quicklime, either in a dry state, or combined with water in particular vessels, so constructed as to bring a large surface of the lime into contact with the gas. This method must naturally be very imperfect, on account of the feeble action of sulphuretted hydrogen upon lime. In proof of this statement, the gas supplied to this metropolis, need only be examined in the following manner:—Collect a four-ounce phial full of the gas, in a wash-hand bason, or other vessel, full of water, in the usual manner, and then plunge into it a slip of paper moistened with a solution of nitrate of silver, or super-acetate of lead. The paper will instantly acquire a brown colour.

A new method of getting rid of the sulphuretted hydrogen gas has been lately resorted to with success; and the facility, cheapness, and expedition, with which this process may be employed in the large way, give reason to believe that it will be highly beneficial to the manufacturer of coal-gas in general. The process consists in passing crude coal-gas, as it is disengaged from coal, through a heated iron cylinder, or other vessel, containing fragments of metallic iron, (the waste clippings of tinned iron will do very well,) or any oxide of iron at a minimum of oxidation: for example,—clay iron-stone, so disposed as to present as large a surface as possible; by this means the sulphuretted hydrogen becomes decomposed by the metallic iron, and the gas is obtained in a pure state. This iron, if in a state of a metal, acquires by this process a crystalline structure, and affords abundance of sulphuretted hydrogen by the affusion of dilute sulphuric or muriatic acid, a proof that it is converted into a sulphuret;—a quantity of sulphuric and sulphureous acid is likewise collected at the extremity of the vessel. The gas thus treated affords no disagreeable odour during combustion, and its purity is attested by its not acting upon the solutions of lead, silver, or any of the white metals.

---

On Tuesday the 16th instant, a deputation of Noblemen and Gentlemen of the county of Cornwall waited upon Dr. Paris, at his house in Dover-street, and presented him with a magnificent Piece of Plate of several hundred pounds value, as a testimony of their grateful sense of his scientific services during his residence amongst them. The inscription is engraved upon a massy silver waiter, and is as follows:—

"To JOHN AYRTON PARIS, M.D. F.L.S. Fellow of the Royal College of Physicians of London, &c. this Plate is inscribed by the Noblemen, Representatives in Parliament, and Gentlemen of the County of Cornwall, in testimony of their grateful sense of his services in originating the Plan, and promoting the Institution, of the Royal Geological Society of the County, which has rendered their home the school of science, and their native riches increasing sources of prosperity."

So handsome a testimony of the talents and industry of a medical practitioner is no less creditable to the general profession of physic, than it is honourable to the scientific individual upon whom it is conferred. One of the great advantages which the labours of Dr. Paris have bestowed upon the county of Cornwall is the introduction of a system of safety to prevent the accidental Explosion of Gunpowder in the Mines, and to which so many have fallen a sacrifice. This is certainly worthy of a physician, whose duty it is, as Dr. Paris has justly observed, to prevent as well as cure the diseases and accidents to which those around him are more immediately exposed; and, by his influence and counsel, to correct the habits, and remove the prejudices, which are most active in producing them.*

Amongst the names of persons who have contributed towards the plate, it is gratifying to notice, Lord De Dunstanville; Lord Exmouth; the Members for the County; Davies Gilbert, Esq. M.P.; Sir Rose Price, Sir Christopher Hawkins, Sir Vyal Vyvyan, Baronets; and a numerous list of other gentlemen.

---

* The reader is referred to the first volume of the Transactions of the Royal Geological Society of Cornwall, which is just published, for an account of this important application of science.

*Water-Spouts.*—The following observations of Captain Thomas Lynn, commander of the East India Company's ship Barkworth, afford a striking corroboration of the statement of the ingenious writer in the *Philosophical Magazine,* Mr. J. H.—viz. that the particles of water ascend upward from the sea, in the phenomenon called a water-spout.

"Barkworth, Dec. 11, 1816, in lat. 4° N. long. 129° E. (having passed through the Siao channel yesterday). At 11 A.M. the officer of the watch, Mr. Dudman, came down and informed me there had been a *whale blowing* close to the ship for several minutes, and that it was continuing to do so. I then, from curiosity, went upon deck, and was surprised to find it was the vortex of a water-spout, within one hundred yards of the ship, on the windward quarter:—ordered a gun to be got ready, by which time it had passed under the stern, within thirty yards of the ship, which afforded us an excellent opportunity of observing this wonderful phenomenon.

"The space it occupied upon the sea was apparently about thirty feet in circumference, and the water so much agitated in the centre as to be quite frothy, *ascending* in a spiral form in *visible particles* like rain, and making a rushing noise about as loud as the blowing of a whale continued, and communicating with a spout* depending from a black cloud over head, gradually passing to leeward, and disappearing about a mile off."

---

Dr. Balfour gives several cases in which the Nitrate of Silver was administered with effect in various disorders.

*Case* 1.—A. applied to me, oppressed with general debility; weakness of the inferior extremities, approaching to paralysis; complete relaxation of the sphincter ani; prolapsus of the gut, accompanied with frequent loss of blood; and a perpetual and copious gleety discharge which had lasted fourteen years. He consulted the late Dr. Beddoes, who guessed the cause of his complaints the moment he saw him walk. Dr. Beddoes asked him if ever he had received an injury in the back? The patient declared positively he never had. But, upon the doctor's insisting on it, he came at last to the recollection of having been struck forcibly on the lumbar vertebræ by the shaft of a gig, a short time before he began to complain. From Drs. Beddoes and King, the last of whom was likewise consulted, he derived no further benefit than what resulted from the application of ligatures to some vessels of the rectum, by which hæmorrhage was checked to a very considerable degree.

Gentle percussion was applied by Dr. B. to the sacrum, the glutei muscles, and the course of the sciatic nerve, with the view of exciting the action of the nerves supplying these parts, and eliciting a transmission of nervous power from the spinal marrow. This

---

"* We could not perceive the communication with the spout, the particles being too minute for the eye to discern much above the sea, but we had no doubt of the fact."

operation was soon followed by increased command and power of the limbs; but had not been repeated above three or four days, when increased discharge of blood from the rectum took place. Till now he had been kept in ignorance of the state of the anus, having his attention directed to the limbs solely. Percussion was given up, and the most powerful liquid astringents were applied to the bleeding surface, which was quite exposed to view, but with little or no effect. The patient requested him "to take up the veins as Dr. King had done," but nothing appeared but an oozing from an extensive surface. Nitrate of silver was now used as an internal astringent, in the quantity of a sixteenth of a grain three times a-day. The sense of fulness which always preceded the discharge, had totally left him, and the discharge itself, of blood, was also greatly diminished,—an effect this which was not, *a priori*, to be expected. With the minute quantity of a sixteenth of a grain three times a-day, sometimes only twice a-day, and sometimes omitted altogether, the gleety discharge had, in two months, ceased almost entirely, the sphincter resumed its functions, and the anus contracted and puckered in the natural way.

*Case* 2.—H. of a consumptive family, complained of general debility; of profuse perspiration on the slightest exertion; of frequent giddiness, especially on turning quickly round; and of a sense of weight in the back part of the head.

An eighth of a grain of nitrate of silver was directed three times a-day; she experienced, in ten days, the beneficial effects of the pills. The dose was increased to one-fourth of a grain, and she continued taking three a-day till about the end of the sixth week. She had now recovered her strength; the sweatings were checked; and she could do her work with perfect ease.

*Case* 3.—W. E. had complained, for nine months back, of pain in the breast, attended with cough and dyspnœa, the latter attacking him in paroxysms almost to suffocation, and found himself getting weaker daily. Dr. B. was called to him owing to his being seized with a most violent stitch under the short ribs, right side. Percussion for two minutes was applied, by which he felt himself greatly relieved, and was enabled to turn himself any way he pleased. In about a quarter of an hour he became sick, and vomited a quantity of greenish yellow substance, very offensive to the taste, and was immediately and entirely relieved of all his complaints. A month after, he was again called in a great hurry, and found him labouring under a tremendous fit of asthma, with a frequent full pulse, and considerable heat of skin. He took fourteen ounces of blood with some relief. Next day he had another paroxysm of asthma, a grain of opium was given. This had the desired effect, not only at this time, but ever afterwards; nor was it ever necessary to increase the dose of opium. One grain, taken when a paroxysm was threatened, completely checked it. Finding, however, that debility, emaciation, and sweating continued, the nitrate of silver was began in the quantity of a sixteenth of a grain twice a-day, or as sweatings occurred. This had the effect of moderating the sweating. The dose was afterwards increased, first to one-eighth of a grain, then to one-fourth, to be taken at any time, by day or night, when menaced by profuse perspiration. The strength was now improved, and he checked the sweatings at any time by one pill; nor was the asthma or cough at all troublesome. He continued the nitrate of silver six months. Every symptom of disease had now disappeared; the patient had recovered flesh, strength, and a healthy appearance: his recovery was considered complete. He was, however, seized with heartburn, which, in a few hours, was succeeded by vomiting of a dark-coloured substance, so thick and tenacious, that it might have been suspended on a stick. Upon this, the patient was again perfectly well; nor did any of his former symptoms return: laxative medicines were ordered, but the same phenomenon recurred repeatedly at short intervals. I had now recourse to the blue pill as an alterative, and with the happiest effects; of these he took five dozen in the course of two months, when he gave over all medicine. Through the course of the succeeding winter, he had few complaints, and used as little medicine. On the approach of spring, however, he was again menaced with a return of heartburn and asthma; symptoms which were immediately checked by emetic tartar, exhibited in the form of pill, and in the quantity of a fourth of a grain at bed-time, as occasion required. If there was any affection of the liver in this case, the blue pill seemed to have a good effect on it; but emetic tartar a better.

*Case* 4.—Mrs. W. had fluor albus, under which she then laboured, and to which she had been occasionally subject for some years. A lotion was prescribed, and the nitrate of silver: she took only two dozen of one-fourth of a grain each, when the discharge disappeared. My patient was very much surprised at the decided effects of the medicine, as she had been, on a former occasion, under the care of an eminent surgeon for the same complaint, and experienced little relief for a great length of time, although she used a great deal of medicine internally.

*Case* 6.—Mr. S. of a strongly-marked scrofulous habit, and having had for many years a copious purulent discharge from the lungs, and difficult respiration, was attacked last summer with what was deemed confirmed consumption. After being greatly reduced, and confined for some time to the house, he came abroad again, still discharging immense quantities of purulent-looking matter, of a bad smell and taste. About the middle of July, he consulted Dr. B. who gave him a quarter of a grain of nitrate of silver four times in the day. Having taken three dozen pills in this way, the sputum was reduced in quantity, and entirely deprived of its bad taste and smell; he now coughed with a vigour, and expectorated with a freedom, to which he had been long a stranger. In the beginning of August he went to the country, carrying along with him twenty dozen of the pills, which he was directed to take as he found them affect him. He returned to town in the beginning of October, much improved in every respect; and, having exhausted his stock, he now took four dozen more, when he dropped them altogether, having no further occasion for medicine.

*Case* 7.—Mrs. S. exhibited the appearance of being far advanced in phthisis; she had laboured for some time under fluor albus; the discharge was copious, extremely acrid, and accompanied with distressing pain in the region of the uterus: she took the nitrate of silver with immediate and good effect. The pain went off in three days, and the discharge was lessened in proportion. In less than a fortnight, she felt her strength considerably improved, and in every respect mended. She continued the medicine about a month, at the end of which she was free from complaint.

*Case* 8.—Mrs. M. had constantly an eruption over her face of a fiery red, and who had not menstruated for several years, became subject to an abscess in a particular spot, within the left *labium pudendi*, of frequent recurrence. She underwent three courses of mercury, but the complaint perpetually recurred. At length, she took nitrate of silver with the happiest effects. Not only did the discharge disappear, and the ulcer heal up kindly, but the whole habit of the patient seemed to undergo a revolution; her face became less red, symptoms of the return of the catamenia began to manifest themselves, and she felt herself better than she had been for several years before.

*Case* 9.—A lady, advanced in life, was seized with a pneumonic affection, accompanied with colliquative perspiration. As soon as the pain of the chest was subdued, which was effected *without blood-letting*, and that the patient could make a full inspiration, nitrate of silver was exhibited. The perspirations were immediately checked, and the strength of the patient was quite restored in a short time.

*Case* 10.—A young man was attacked with slight obtuse pain in his chest, which ended in colliquative perspiration. This was, indeed, his only complaint; and the nitrate of silver removed it entirely in a very few days.

Dr. B. has also exhibited nitrate of silver in obstinate gleets from gonorrhœa; and, in some instances, with perfect success, after all the usual remedies had been tried in vain. One gentleman had been repeatedly under his care for gonorrhœa, which in every instance was difficult of cure. The last time nitrate of silver was given after the inflammatory symptoms were subdued, and with immediate good effect. Not only was the discharge quickly dried up, but the patient described himself as having acquired a tone and vigour to which he was formerly a stranger. It was with difficulty also that this patient was persuaded to give over the medicine.—*Med. Chir. Journ.*

---

*A Female Dwarf.*—A girl is now publicly exhibited in Paris, who is nearly eight years of age, and only twenty-one inches in height, which is about that of a full-grown fœtus: the weight is also proportionate, being eight pounds and a half. It is said, that, at the period of birth, she weighed a pound and a half, and was eight inches in length. It varies in the relative dimensions of the different parts of the body in this respect,—that the middle line is situated at two-thirds of the distance from the umbilicus to the eminence of the pubis; while, in the fœtus, it is nearly equidistant between the umbilicus and sternum. The bones are well-formed; she has lost several of her first teeth, and some of the permanent incisors are making their appearance. The muscles are firm and powerful; and their distribution can be clearly distinguished beneath the skin; but there is a constant irregularity of muscular action over the greater part of the body. The organs of sense are perfect, except the eyes, which are myopic: the nutritive functions are healthy. Her parents, a brother, and three sisters, are all persons of ordinary stature.—*Gaz. de Santé.*

---

*On the Use of Tar-Vapour in Asthma.*—The Tar Vapour has been employed with advantage by Mr. Wansbrough, in a lady who was attacked about twelve months since with spasm in the chest, and difficulty of breathing; which increased, in the course of fifteen or twenty minutes, to a pitch that threatened instant suffocation. The attack commenced at ten o'clock at night, on the 21st of March. Mr. W. observes, "The poor creature was supported in bed by pillows, completely erect, any other posture increasing the dyspnœa. Her countenance exhibited the acmé of distress: her eyes appeared ready to burst from their sockets; her nostrils were distended; her mouth was extended; her hands clasped in agony. She was incapable of utterance, and every inspiration was performed with distressing effort, accompanied by a loud and deep sound. The case altogether indicated a necessity for the most prompt measures, in order to prevent suffocation. Her pulse was exceedingly rapid, small, and hard. I immediately opened a vein in the arm, and having made a large orifice, twelve ounces flowed in five minutes, but with only trifling mitigation of symptoms. I obtained a funnel, which, with a pot, formed a good substitute for an inhaler: through this I passed the vapour of hot water into the lungs, which my patient inhaled most eagerly: after two or three inspirations, in a few minutes she appeared relieved; and I had the pleasure to perceive a decided alteration for the better in the course of an hour; yet not to the extent of my wishes. As there still existed considerable distress in the act of inspiration, I directed the inhalation to be continued, constantly renewing the heat by the addition of hot water as soon as the last began to cool; and exhibited ten grains of calomel, with a saline purgative mixture, every fourth hour.

I saw her again at eight o'clock the next morning; the difficulty of breathing still continued to distress her much, although it was considerably relieved from what it was when I first saw her. The blood drawn did not exhibit the slightest appearance of buff. She was now able to answer such questions as I put to her, sufficiently to be understood, although in unconnected words, not exceeding monosyllables; by which I could collect, that she had been subject to these attacks upon exposure to cold; that she had often a cough, with little expectoration; and always, according to her statement, shortness of breath. Having made out the case to be chronic asthma, I ceded my patient to the medical practitioner, whose patient she properly was. This gentleman applied a blister to the chest, and exhibited antispasmodics, by which she was benefited; but did not recover her strength sufficient for walking, even a few yards, for some months afterwards.

Two months ago, I was again called to the same patient; the former agony and distress were visible in her countenance: she had been in that situation about an hour before I saw her. Instead of adverting to the venesection and the vapour of hot water, I had now recourse to the vapour of tar; and never did I witness any thing equal to the effect which this simple, yet powerful, remedy produced: she had no sooner inspired it than she was relieved. In the space of less than two minutes the sonorous inspiration had declined; and in half an hour from the commencement of the process, my patient exclaimed, "O! Sir, I am in Heaven!"—She recovered rapidly in the course of the same day; and when I visited her in the evening, she complained of no inconvenience or pain, save debility and a little head-ach. The following morning was ushered in by a slight relapse, owing, I supposed, to the vapour being suspended during the night. Considerable nausea, with strong disposition to vomit, yielded to the exhibition of twenty grains of ipecacuanha, which relieved the stomach of a quantity of black matter, highly impregnated with the smell of tar. The vapour was continued without intermission, and she took some trifling antispasmodic, with aperient medicine, for three days; when she declared herself as well as ever she was. The method employed in exhibiting the vapour, was similar to that recommended by Dr. Chrichton.—*Med. Chir. Journ.*

---

*On the Non-contagious Nature of the Yellow Fever.*—Messrs. Halle, Leroux, and Chaussier, in a report to the French government, on the possibility of the yellow fever being imported into France, express themselves as follows:—While the contagious or non-contagious nature of the malady in question remains uncertain, it is advisable for government to adopt measures founded upon the presumption of its communicable nature: but there were many instances of the fever breaking out during the enforcement of quarantine regulations, while the season was favourable to its production, and while the ship in which it appeared contained putrescent animal matter. In this case, the very act of quarantine was a cause of the disease, which might have been prevented by discharging and purifying the vessel; a fact, indeed, which was proved in the Marseille, at Columbia, in 1802, and other ships in 1804; for, had the malady, which made its appearance, been of a really contagious nature, how could it have been arrested, while the vessel still remained at Columbia?

In America, while the fever raged both in the islands and on the continent, many vessels that had put in at several American ports, afterwards sailed for French ports, without having been subjected to any quarantine restrictions, and no single instance occurred of the importation into France of the disease. In the United States, individuals indiscriminately and fearlessly exposed themselves to contact with those who were affected with yellow fever, even lying in their beds either before or after their death, wearing their clothes, opening their bodies, inoculating themselves with their blood, saliva, and ejected contents of their stomach, and still continuing free from the malady, provided they were out of the sphere of infection. "*S'ils etaient hors de la sphère d'activité de l'infection.*"

Among several American ships of war in which the yellow fever broke out, the Ganges, the Warren, the Delaware, the General Green, were principally affected. Upon the return of these vessels, their several sick men were carried to the hospitals with all their clothes and effects; and, notwithstanding this want of precaution against its visits, the disease did not show itself upon a single inhabitant. In like manner Lind observed, in the Haslar Hospital, near Portsmouth, that, although many were received into that hospital, with the yellow fever upon them, and although it was impossible to sequester these individuals from communication with others, that nevertheless the disease was not known to propagate itself in a single instance. In fact, although an abundance of testimony proves the frequent breaking out of the disorder in question, in vessels which go from this to the other side of the Atlantic, we never find the recrossing from America to Europe productive of the same effect.

When cold succeeds to heat, the yellow fever invariably declines and dies away, which would not be the case were it really of a contagious nature; and he repeats, as a conclusion from the whole of the enquiry, that it is perfectly chimerical to conceive either the transportable nature of the miasmata of those fevers, or their contagious properties.—*Lond. Repository.*

---

*Monstrous Fœtus.*—An official account has been published in France of a monstrous fœtus, or rather of two fœtusses, produced by the wife of a labourer at Montfort l'Amaury. The disposition of their bodies bears a strict resemblance to that of the two girls described by Buffon; both perfectly formed as far downwards as the last vertebræ of the loins, where they unite, and the union is continued throughout the whole length of the sacrum, one bone being apparently common to both, which terminates in a single coccyx; with the exception of the last-mentioned bone, there are two distinct and perfect pelves, from which four well-formed inferior extremities have their origin. In the middle space of the upper part of the four thighs, are the genital organs, consisting of one scrotum with four testicles, and one penis situated in the middle of the scrotum. The anus is situated between the scrotum of the coccyx. The urine and fæces were freely evacuated. One of the bodies was an inch and a half longer, and much stronger, than the other, which was very feeble, and of a livid colour.—The woman was attended by a midwife, who states, that the body described as the strongest, presented first naturally; and, after a very severe labour, of some days' continuance, both were expelled.

She could not explain in what way the second was expelled, but it probably was doubled,—the head coming away last. There was only one placenta, having a funis divided into two portions: one of the usual size, going to the strongest of the fœtusses, the other was very slender. They were exposed for the gratification of public curiosity for several days, when they both died at the same instant. The father, wishing to make them an object of gain, would not permit an examination that, by altering the external appearance, could interfere with his design. All that has been ascertained of their internal formation is, that the inferior extremities of the two colons unite to form a rectum common to both bodies, and the necks of two bladders join in a similar manner to constitute a single urethra.—*Gazette de Santé.*

---

A gentleman has invented a perfectly novel apparatus (see the advertisement) for making the Labels used by Apothecaries and Chemists, in a neat, ornamental manner, by machinery.

---

Died, at his house in Hatton Garden, aged 62, Dr. Joseph Adams, many years Physician to the Hospital for the Small-Pox, for Inoculation, and for Vaccination at Pancras, and to the New Finsbury or Central Dispensary. He was the author of several works, and well known to the medical profession.

---

### REPORT OF DISEASES.

The beginning of the month having been unusually warm, and succeeded by rain, cholera and bilious diarrhœa have been frequent. They have not, however, excepting in one instance of the former, been attended with any difficulty in their removal. Dilution, followed by magnes. carbon. in aq. menth. virid. alone, or conjoined with a few drops of tinct. opii. have been all that have been required in the former; and, in the latter, a purgative succeeded by mistur. cretæ have absolved the cure. In the exception spoken of above, opii. gr.¼, in forma pilulæ exhibited every two hours, with a large blister applied to the scrobiculus cordis, were found soon to restrain the incessant vomiting which took place, and to subdue the complaint. A case of typhus mitior, which terminated fatally in a female of middle age, is the only one of fever that has occurred since our last report. Head-achs, and some of them severe, have been common; and, as all appeared connected with vascular fullness, topical bleeding and brisk purgatives were had recourse to: they generally afforded relief, and, if the ingesta were at the same time attended to, seldom recurred. Three cases, one occasioned by the lime used in purifying the gas employed in lighting the streets, the others of porrigo scutulata, and impetigo figurata, it may not be uninteresting to relate.

A strong middle-aged man, employed in the Dorset-street Gas Works, after having removed a considerable quantity of lime through the water of which the gas had passed, was seized with vertigo and paralysis of the superior extremities. We saw him three days after the attack: his eyes were blood-shot; he complained of great head-ach and thirst: his arms were swollen and stiff, and he was unable to raise them from his sides. As he had been bled from the arm freely, he was ordered to be cupped in the neck, to take a strong cathartic every other morning, and to observe a strict antiphlogistic regimen. He recovered the use of his arms in four days; and, in a week was enabled to resume his employment.

Porrigo scutulata occurred in a boy about eight years of age. The scalp was nearly incrustated; the disease had been of nine months' continuance, and had resisted, as we were informed, the various applications which had been made use of. The head was directed to be shaved twice a week, to be well washed every morning with soap-lather, the ung. sulph. cum pice to be applied night and morning, and to take hydrarg. submuriat. gr. ſs. omni nocte. By persevering steadily in this plan for a month, the disease was perfectly removed. We may be allowed to remark here, that, from pretty extended experience, we have found the unguent. sulph. cum pice, of all applications employed in this disease, the most to be depended on. It has, in our hands, seldom or never failed.

The subject of impetigo figurata was a young lady, aged twenty-three, of a fair complexion and sanguine temperament, and not subject previously to any cutaneous disease. In February last, she consulted the Reporter, for an eruption on the left hand: it was not larger than a shilling, and had made its appearance only three days before. As she was otherwise in perfect health, she was only ordered to apply a little unguent. zinci to it, night and morning. It getting no better at the end of a fortnight, the ointment was changed for the unguent. calcis. hydrarg. alb. This, however, was found to be too stimulating, and it was dressed with unguent. super-acet. plumbi. The eruption now beginning to extend itself over the hand, she was ordered to abstain from animal food and fermented liquors, to take magnes. sulph. ℥ſs. in aq. menth. virid. alternis auroris, pilul. submuriat. hydrarg. gr. v. mane nocteque, and, after bathing the part with warm milk and water twice a-day, to cover it with an ointment composed of equal parts of unguent. zinci. and unguent. super-acet. plumbi. The disease continuing to spread, and appearing on the other hand, and as the inflammation was considerable, and the pain and itching intolerable, a bread-and-milk poultice, after the bathing, was applied morning and evening. This afforded great relief, lessening the inflammation, pain, and itching, and disposing the parts to heal. This application, the only one that had been the least serviceable, was continued for a month, during which time she took only her aperient mixture, the pills having been omitted from their appearing to stimulate. The disease having now continued three months nearly, and the poultice having lost its effect, she went into the country, and tried, by the advice of a female friend, a fomentation made from a decoction of elder flowers. This gave her more ease than any application which had hitherto been employed, and effected a cure of this obstinate disease in the short space of a fortnight.

### METEOROLOGICAL JOURNAL,

By Messrs. William Harris and Co. 50, Holborn, London.

*From the 20th May to the 19th June, inclusive.*

| Day of Month. | Moon. | Rain gauge | Therm. | | | Barom. | | De Luc's Hygrom. Dry | | De Luc's Hygrom. Damp | | Wind. | | Atmospheric Variation. | | |
|---|---|---|---|---|---|---|---|---|---|---|---|---|---|---|---|---|
| May | | | | | | | | | | | | | | | | |
| 20 | ○ | | 51 | 61 | 45 | 30·03 | 30·12 | | | 10 | 12 | NE | NE | Fine | | |
| 21 | | | 50 | 58 | 43 | 30·12 | 30·18 | | | 10 | 9 | NE | NE | Clo. | Fine | Clo. |
| 22 | | | 49 | 54 | 41 | 30·19 | 30·24 | | | 7 | 9 | NE | NE | Fine | | Clo. |
| 23 | | | 49 | 57 | 41 | 30·17 | 30·12 | | | 5 | 6 | NE | NE | Clo | Fine | |
| 24 | | | 48 | 56 | 42 | 30·17 | 30·21 | | | 2 | 2 | NE | NE | Fine | | |
| 25 | | | 50 | 58 | 45 | 30·25 | 30·25 | | | 1 | 1 | NE | NE | Fine | | |
| 26 | ○ | | 49 | 60 | 48 | 30·29 | 30·30 | | 0 | 1 | 0 | NE | NE | Fine | | |
| 27 | | | 52 | 59 | 48 | 30·28 | 30·23 | 0 | 0 | 0 | 0 | NE | N | Fine | | |
| 28 | | | 52 | 61 | 47 | 30·20 | 30·17 | 0 | 0 | 0 | 0 | NE | NE | Fine | | |
| 29 | | | 53 | 61 | 48 | 30·12 | 30·13 | 1 | 1 | | | E | E | Clo. | Fine | |
| 30 | | | 55 | 63 | 50 | 30·09 | 30·00 | 0 | 0 | 0 | 0 | N | N | Fine | | |
| 31 | ◑ | ,04 | 57 | 65 | 51 | 29·99 | 29·97 | | | 7 | 9 | E | N | Fine | | |
| June | | | | | | | | | | | | | | | | |
| 1 | | | 57 | 68 | 53 | 29·90 | 29·90 | | | 9 | 11 | N | SW | Fine | Clo. | Rain |
| 2 | | | 59 | 68 | 54 | 29·91 | 29·98 | 0 | 5 | 0 | | SW | S | Fine | | |
| 3 | | | 58 | 66 | 54 | 30·00 | 30·00 | 2 | 0 | | 0 | SW | N | Fine | | |
| 4 | | | 59 | 69 | 56 | 30·03 | 30·07 | 7 | 3 | | | NE | NE | Fine | | |
| 5 | | | 60 | 70 | 58 | 30·18 | 30·22 | 5 | 3 | | | NE | N | Fine | | |
| 6 | | | 61 | 70 | 56 | 30·29 | 30·35 | 5 | 5 | | | E | E | Fine | | |
| 7 | | | 61 | 75 | 57 | 30·39 | 30·40 | 2 | 1 | | | NE | N | Fine | | |
| 8 | | | 63 | 78 | 57 | 30·37 | 30·33 | 0 | 0 | 0 | 0 | E | NE | Fine | | |
| 9 | | | 63 | 77 | 57 | 30·30 | 30·27 | | | 9 | 11 | E | NE | Fine | | |
| 10 | | | 63 | 77 | 59 | 30·21 | 30·19 | | | 13 | 7 | NNE | N | Fine | | |
| 11 | ○ | | 65 | 78 | 58 | 30·15 | 30·10 | 0 | 0 | 0 | 0 | E | ENE | Fine | | |
| 12 | | | 67 | 80 | 61 | 30·07 | 29·98 | | | 4 | 5 | SE | SW | Fine | | Clo. |
| 13 | | | 69 | 80 | 59 | 29·91 | 29·90 | | | 5 | 7 | SSW | WSW | Fine | Rain | Fine |
| 14 | | ,02 | 63 | 70 | 60 | 29·98 | 30·04 | | | 10 | 10 | SE | E | Clo. | Showery | Clo. |
| 15 | | | 64 | 69 | 59 | 30·04 | 29·95 | | | 11 | 10 | SSW | SW | Clo. | Fine | |
| 16 | | | 65 | 73 | 59 | 29·94 | 29·85 | | | 11 | 15 | WSW | S | Fine | | |
| 17 | | | 61 | 72 | 56 | 29·79 | 29·74 | | | 15 | 10 | S | S | Clo. | Rain | |
| 18 | ○ | ,05 | 59 | 69 | 57 | 29·74 | 29·73 | | | 10 | 10 | SW | NW | Fine | Showery | |
| 19 | | ,07 | 59 | 67 | 53 | 29·72 | 29·63 | | | 10 | 10 | SW | SW | Fine | Rain | |

The quantity of rain fallen in the month of May is 1 inch and 19-100ths.

